# Genome Characterisation of Enteroviruses 117 and 118: A New Group within Human Enterovirus Species C

**DOI:** 10.1371/journal.pone.0060641

**Published:** 2013-04-02

**Authors:** Antonio Piralla, Cristina Daleno, Alessia Scala, David Greenberg, Vytautas Usonis, Nicola Principi, Fausto Baldanti, Susanna Esposito, for the CAP-PRI Study Group

**Affiliations:** 1 Molecular Virology Unit, Fondazione Istituto Di Ricovero e Cura a Carattere Scientifico Policlinico San Matteo, Pavia, Italy; 2 Pediatric Clinic 1, Università degli Studi di Milano, Fondazione Istituto Di Ricovero e Cura a Carattere Scientifico Ca' Granda Ospedale Maggiore Policlinico, Milan, Italy; 3 The Pediatric Infectious Disease Unit, Soroka University Medical Center, Beer-Sheva, Israel; 4 Vilnius University Clinic of Children's Diseases, Vilnius University, Vilnius, Lithuania; The University of Hong Kong, China

## Abstract

The more than 120 genotypes of human enteroviruses (HEVs) reflect a wide range of evolutionary divergence, and there are 23 currently classified as human enterovirus C species (HEV-C). Two new HEV-C (EV-C117 and EV-C118) were identified in the Community-Acquired Pneumonia Pediatric Research Initiative (CAP-PRI) study, and the present paper describes the characterisation of the complete genome of one EV-C117 strain (LIT22) and two EV-C118 (ISR38 and ISR10) strains. The EV-C117 and EV-C118 5′UTR sequences were related to those of EV-C104, EV-C105 and EV-C109, and were slightly shorter than those of other HEV A-D species. Similarity plot analyses showed that EV-C117 and EV-C118 have a P1 region that is highly divergent from that of the other HEV-C, and phylogenetic analyses highly supported a monophyletic group consisting of EV-C117, EV-C118, EV-C104, EV-C105 and EV-C109 strains. Phylogenetic, Simplot and Bootscan analyses indicated that recombination was not the main mechanism of EV-C117 and EV-C118 evolution, thus strengthening the hypothesis of the monophyletic origin of the coding regions, as in the case of other HEV-C. Phylogenetic analysis also revealed the emergence of a new group within HEV-C that is divided into two subgroups. Nucleotide and amino acid identity in VP1 sequences have been established as useful criteria for assigning new HEV types, but analysis of the complete P1 region improves resolution.

## Introduction

Human enteroviruses (HEVs) belong to the *Picornaviridae* family and cause a wide range of clinical conditions, ranging from asymptomatic or mild infections to more severe diseases such as acute hemorrhagic conjunctivitis, aseptic meningitis, severe community-acquired pneumonia (CAP), gastroenteritis, acute flaccid paralysis, myocarditis, and neonatal sepsis-like disease [1]. HEVs are non-enveloped viruses characterised by a single positive-strand RNA genome that consists of approximately 7,500 nucleotides (nt) and contains a single open reading frame (ORF) flanked by untranslated regions (UTRs) at each end. The ORF encodes a polyprotein that is post-translationally cleaved to yield four structural (VP4, VP2, VP3 and VP1) and seven non- structural proteins (2A, 2B, 2C, 3A, 3B, 3C and 3D) [2].

Enterovirus typing is based on comparing the sequences encoding the VP1 capsid protein: viruses of different genotypes have <75% nucleotide identity, and <85% amino acid identity [3], [4]. HEVs are currently divided into four species (HEV-A to HEV-D) depending on their sequence similarities and biological properties. Twenty-three types of HEV-C have so far been identified (www.picornaviridae.com/enterovirus/hev-c/hev-c.htm), a number of which have been identified in the last decade. EV-C104 and EV-C109, which were detected in patients with respiratory syndrome in Switzerland and Nicaragua [5], [6], are distinct among the HEV-C species and have been found to circulate worldwide [7]–[11]. In 2010, EV-C105 and EV-C116 were detected in patients with gastroenteritis in the Republic of Congo and Sakhalin Island [12], and, other EV-C105 strains have been detected in subjects with respiratory disease in Peru [13]. The most recently discovered HEV-C strains, EV-C117 and EV-C118, were detected during the course of the Community-Acquired Pneumonia Pediatric Research Initiative (CAP-PRI) study in the winter of 2010-2011 [14]–[17].

The aims of this study were: i) to characterise the complete genome of one EV-C117 (LIT22) strain and two EV-C118 strains (ISR10, and ISR38); and ii) to investigate the relationship between these new viruses and other HEV-C strains.

## Materials and Methods

### Ethics Statement

The study was approved by the Ethics Committee of the participating Centers: Università degli Studi di Milano, Milan, Italy; The Pediatric Infectious Disease Unit, Soroka University Medical Center, Beer-Sheva, Israel; Vilnius University Clinic of Children's Diseases, Vilnius, Lithuania; Fondazione IRCCS Policlinico San Matteo, Pavia, Italy. It was conducted in accordance with the standards of Good Clinical Practice according to the principles of the Declaration of Helsinki; the children's parents or legal guardians gave their written informed consent before the children were enrolled.

### CAP-PRI study

The CAP-PRI study was a prospective community-based study designed to evaluate the prevalence of respiratory virus infections in children admitted to seven pediatric hospitals in Italy, Israel, Greece, Portugal, Lithuania and Romania because of radiographically confirmed CAP. Study enrolment began on 1 November 2010 and ended on 31 March 2011. A nasopharyngeal sample was collected from all of the children upon admission using a flexible pernasal flocked swab that was immediately placed in a mini-tube containing 1 mL of universal transport medium (UTM-RT Kit Cat. No. 360c, Copan Italia, Brescia, Italy). The samples were stored at 4°C in the hospital laboratory until they were sent in a refrigerated package to the central laboratory (Pediatric Clinic 1, Department of Pathophysiology and Transplantation, University of Milan, Italy), where they were aliquoted and stored at −80°C.

### Specimens nucleic acid extraction, and enterovirus identification

Viral nucleic acids were extracted from the nasopharyngeal swabs by means of a Nuclisens EasyMAG automated extraction system (Biomeriéux, Craponne, France), and the extracts were tested for respiratory viruses using the RVP Fast assay in accordance with the manufacturer's instructions (Luminex Molecular Diagnostics Inc., Toronto, Canada). The samples that were positive for enterovirus/rhinovirus were retested in order to identify the rhinovirus. Phylogenetic analyses of the VP4/VP2 region showed that some nucleotide sequences belonged to the HEV species. The complete VP1 of two of these samples was submitted to the *Picornaviridae* Study Group (www.picornastudygroup.com), compared with other enterovirus sequences, and named as proposed new enterovirus types EV-C117 (accession number JQ446368) and EV-C118 (JQ768163).

### Complete genome sequencing

We analysed one EV-C117 strain (LIT22) and two EV-C118 strains (ISR38 and ISR10). The complete genome sequence was obtained using degenerate primers designed by means of the multiple alignment of the EV-C104 and EV-C109 genomes available in GenBank, and additional primers designed on the basis of the first and subsequent rounds of sequencing in accordance with the primer walking method (the primer sequences are available upon request). Briefly, the eluted RNA was transcribed into cDNA using Moloney's murine reverse trascriptase (MMLV-RT, Invitrogen, Monza, Italy) and random hexamers for one hour at 37°C. The MMLV-RT was subsequently denatured at 70°C, and the PCR was carried out in a final volume of 50 µL containing the virus-specific oligonucleotide primers (0.2 µM each), 2 U AmpliTaq Gold 360 DNA polymerase (Applied Biosystems, Foster City, CA), 1× reaction buffer, 0.2 mM of each dNTP, 2 mM MgCl_2_, and 2 µL of c-DNA template.

The PCR products were gel-purified using the Wizard SV Gel and PCR Clean-Up System (Promega, Milan, Italy), and then sequenced in both directions using the same forward and reverse primers as those used in the PCR. The nucleotide sequences were obtained by means of automated DNA sequencing using an ABI PRISM 3730 genetic analyser (Applied Biosystems). The 5′ and 3′ segment sequences were determined using the 5′–3′ rapid amplification of cDNA ends (RACE) in accordance with the manufacturer's instructions (Roche, Mannheim, Germany), and the sequences were assembled using Sequencher software, version 4.6 (Gene Codes Corporation, Ann Arbor, USA).

### Genome analyses

The sequences were aligned using the ClustalW program integrated in the MEGA package, version 5.0 [18]. The best-fitting nucleotide substitution model was estimated using a hierarchical likelihood ratio for each of the analysed genome regions. The phylogenetic tree was reconstructed using the maximum likelihood method and parameters selected by the model test program in the MEGA program. Branch support was assessed by means of bootstrap analysis with 1000 replicates, and a bootstrap value of 70% was used as the cut-off point for cluster analysis. The similarity plot and recombination analyses were made using SimPlot software, version 3.5.1 [19]. Bootscan analysis was used to investigate recombination events within the EV-C117 and EV-C118 genomes. The ORF polyprotein sequence was analysed using the NetPicoRNA server, version 1.0 (http://www.cbs.dtu.dk/services/NetPicoRNA), in order to predict the picornaviral protease cleavage sites (3C^pro^, 2A^pro^ and autocatalytic sites). The RNA secondary structure was predicted using the RNAalifold [20] and Mfold servers [21], whereas the VP1 secondary structure was investigated using the Jpred3 server [22].

### Data collection and nucleotide sequence data set

The complete genome sequences of EV-C117 strain LIT22, EV-C118 strain ISR10 and EV-C118 strain ISR38 were deposited in GenBank and received the accession numbers JX232682, JX961708, and JX961709, respectively. These sequences were compared with 21 complete genomes of other HEV-C strains: EV-C104 strain AK11 (AB686524); EV-C104 strain CL-12310945 (EU840733); EV-C109 isolate NICA08-4327 (GQ865517); EV-C104 strain Pav262-11228 (JX982259); EV-C105 (JX514943); EV-C116 (JX514942); CV-A19 strain 8663 (AF499641); CV-A22 strain Chulman (AF499643); CV-A1 strain Tompkins (AF499635); EV-C96 strain BAN00-10488 (EF015886); EV-C99 (EF555644); CV-A21 strain Kuykendall (AF546702); CV-A24 (D90457); CV-A13 strain Flores (AF499637); CV-A11 strain Belgium-1 (AF499636); CV-A17 strain G12 (AF499639); EV-C102 (EF555645); CV-A20 strain IH35(AF499642); poliovirus type 3 strain Leon (K01392); poliovirus type 1 strain Mahoney (V01148); and poliovirus type 2 strain Sabin (X00595).

## Results

### Genome description

The genome lengths of the EV-C117 and the two EV-C118 strains were respectively 7,362 nt and 7357 nt. Both genomes had a typical enterovirus organisation, with a coding region of 2,206 codons translated into four structural (VP4, VP2, VP3 and VP1) and seven non-structural proteins (2A, 2B, 2C, 3A, 3B, 3C and 3D). The genome of EV-C118 strain ISR10 differed from that of EV-C118 strain ISR38 in 57 nucleotide positions (0.77% divergence) scattered in the 5′UTR, and the P1, P2 and P3 regions. A single amino acid change (0.04% divergence) was observed in the P2 region.

**Figure 1:**
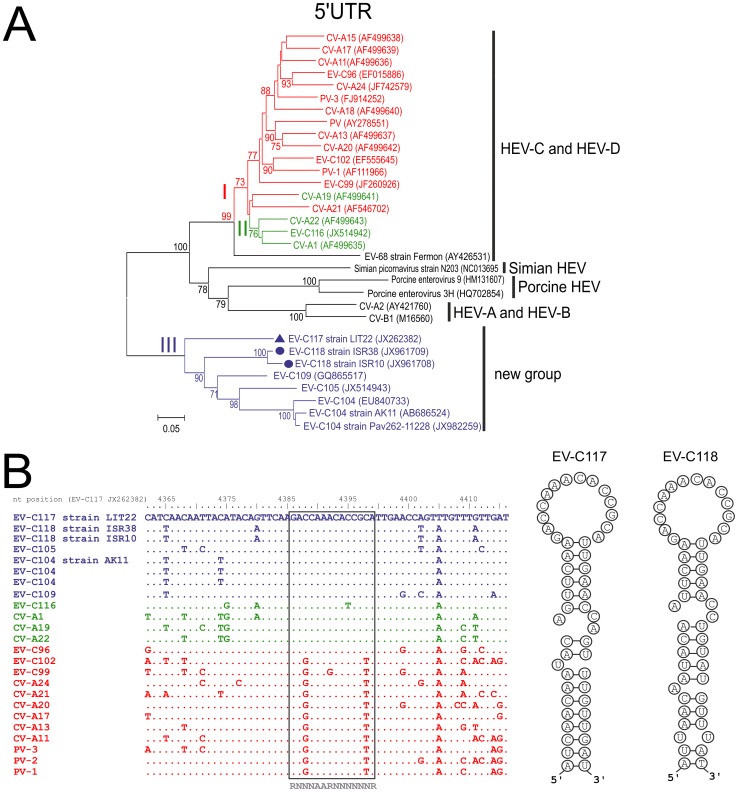
A phylogenetic tree based on 5′UTR sequences and showing the genetic relationships between enteroviruses (A). Nucleotide alignment of the 2C region containing the *cre* element in HEV-C (B).

The 5′UTR consisted of 666 nt in the EV-C117 genome and 671 nt in the EV-C118 genome. The phylogenetic tree constructed on the basis of the 5′UTR sequences showed that EV-C117 and EV-C118 clustered in a group that was different from that of the other HEV-C prototype strains (Fig. 1A), and consisted of EV-C104, EV-C105, EV-C109, EV-C117 and EV-C118 strains. The *cis*-acting replication element (CRE) was detected in the region of protein 2C (Fig. 1B), and had the classic hairpin structure. Both EV-C117 and EV-C118 had the cysteine-rich sequence motif (CX_2_CX_8_CX_4_C) in the 2C protein, the RNA-binding domain KFRDI in the 3C protein, and the two catalytic triads of H_20_-D_38_-C_109_ in the 2A protein and H_40_-E_71_-C_147_ in the 3C protein. The 3D NTP-binding motif (S/T)KVEQGKS was observed with one change in the EV-C117 genome (KVEAGKS), and with three changes in the EV-C118 genome (SKIKAGKS).

**Figure 2:**
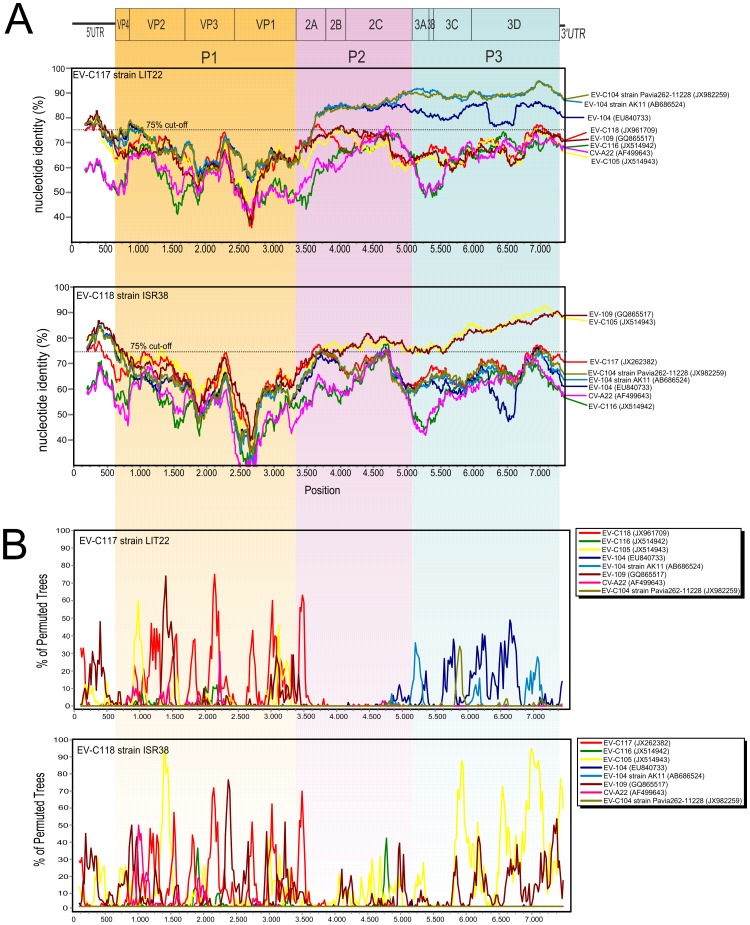
Similarity (A) and Bootscan plot of the complete EV-C117 and EV-C118 genome using a sliding window of 200 nt moving in 20 nt steps (B).

Similarity plot analysis showed that the P1 regions of EV-C117 and EV-C118 were highly divergent from those of the other HEV-C (Fig. 2A). In the P2 and P3 regions, EV-C117 was closely related to EV-C104 strains, whereas EV-C118 was closer to EV-C109 and EV-C105 (Fig. 2A). Bootscan analysis did not reveal any evidence of recombination events in either the EV-C117 or EV-C118 coding region (Fig. 2B).

### Predicted VP1 structure

**Figure 3:**
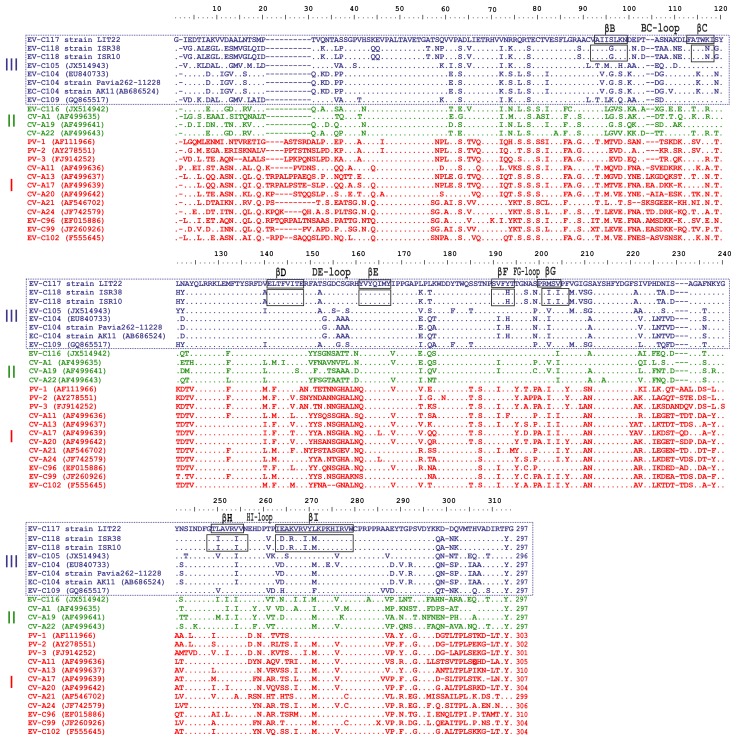
Alignment of the VP1 amino acid sequences of EV-C117, EV-C118 and the HEV strains. The β-sheets are identified by black boxes. The gaps are indicated by a dash (-) and the conserved amino acid residues by a dot (.).

As shown in Figure 2A, EV-C117 and EV-C118 VP1 nucleotide identity was <75% that of the other HEV-C. Figure 3 shows the alignment of the VP1 amino acid sequences of EV-C117, EV-C118 and the other HEV-C genotypes. The VP1 region of both EV-C117 and EV-C118 was 297 amino acids in length. Eight β-sheets (βB to βI) were predicted in the VP1 sequences by the Jpred3 server. Both the EV-C117 and EV-C118 VP1 regions conserved 181 out of 297 residues (60.9%). Inside the motifs corresponding to the β-sheets, there were fewer amino acid changes in the EV-C117 sequence than in the EV-C118 sequence. The greatest variability between EV-C117 and EV-C118 was observed in the region of the BC loop, and in the N- and C-terminus of the VP1 protein.

### Phylogenetic relationship

**Figure 4:**
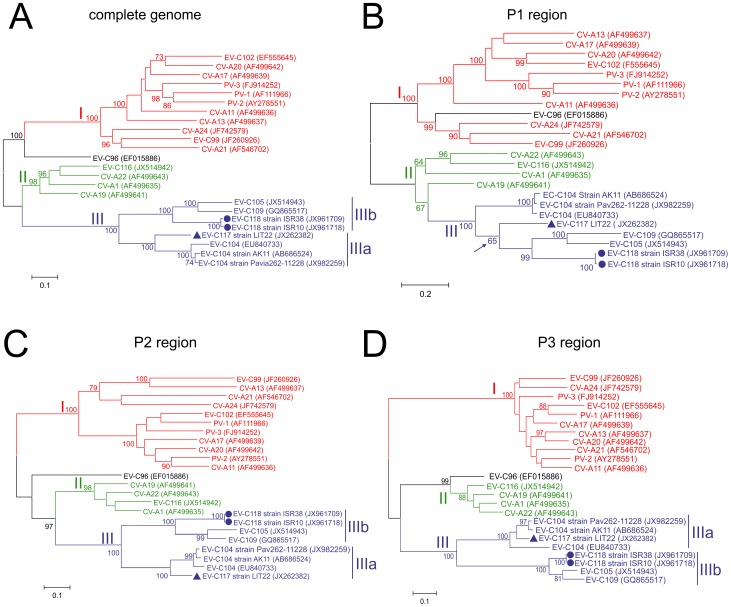
Maximum likelihood trees based on EV-C117, EV-C118 and HEV-C virus nucleotide sequences. Separate tress were constructed for the complete genome (A), and the P1 (B), P2 (C), and P3 regions (D).

**Figure 5:**
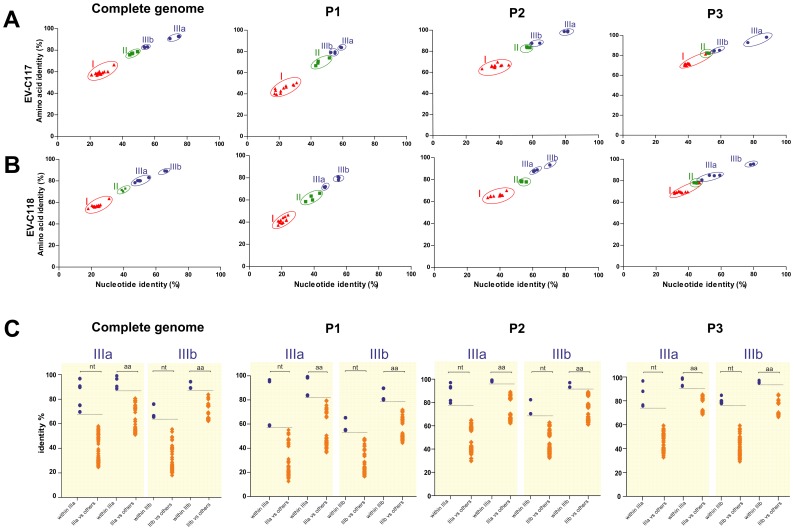
Analysis of sequence relationships by plotting the amino acid sequence identity *vs* nucleotide sequence identity of EV-C117 (A) and EV-C118 (B) against those of the other HEV-C types. The pairwise comparisons with group I, group II and group III sequences are respectively labelled by red triangles, green squares, and blue circles. (C) Nucleotide and amino acid pairwise identities plotted for each HEV-C pairwise sequence within group IIIa and IIIb (filled circles), and against the other groups (red diamonds).

Separate maximum-likelihood nucleotide sequence phylogenies were constructed for the complete genome, and the P1, P2 and P3 regions of EV-C117, EV-C118 and the other HEV-C (Fig. 4). As shown in Figures 4 and 5, the complete genome phylogeny included three distinct groups (group I, II, and III) with bootstrap support of >98% (Fig. 4A). A monophyletic group consisting of EV-C117, EV-C118, EV-C104, EV-C105 and EV-C109 strains was highly supported and, in this group, the length of the internal branches and the high bootstrap values of the nodes suggested two distinct subgroups (IIIa and IIIb); Figure 4A shows that the EV-C117 strain was more closely related to the EV-C104 strains (subgroup IIIa), whereas the EV-C118 strains were more closely related to the EV-C105 and EV-C109 strains (subgroup IIIb).

In the P1 region tree, the position of the EV-C117 strain was partially unresolved and there was a low bootstrap value for specific bipartition (Fig. 4B), whereas the EV-C118 strains grouped with the EV-C109 and EV-C105 strains. The P2 region tree appeared to correspond to the complete genomes tree, with a similarly resolved topology (Fig. 4C). The P3 region tree revealed phylogenetic incongruence within the EV-C104 strains (Fig. 4D) as EV-C117 grouped with two EV-C104 strains, but not with a third (Fig. 4D). Conversely, the position of EV-C118 in the P3 region tree was resolved as in the other trees.

### Pairwise sequence identities

The nucleotide sequence identity of EV-C117 and EV-C118 with the other HEV-C types was plotted against their amino acid sequence identity (Figs. 5A and 5B). As shown in Figure 5, four distinct groups could be identified in the complete genome pairwise identity plots, as observed in the corresponding phylogenetic tree (Fig. 4A), and the same was true of the P1, P2 and P3 region plots.

In order to resolve the intra- and inter-group variability further, we analysed the nucleotide and amino acid pairwise distance within groups IIIa and IIIb, and in relation to the other HEV-C groups (Fig. 5C), by generating plots for the complete genome, and the P1, P2 and P3 regions. Groups IIIa and IIIb were distinct from the other groups in all of the plots. However, as previously observed in the phylogenetic analysis, the P1 region sequences in group IIIa were less different than those in the other groups. This finding mirrors the unresolved topology of the EV-C117 strain shown in Figure 4B. Finally, four different groups were identified on the basis of the phylogenetic analyses and the similarities described above.

## Discussion

Over the last ten years, a number of new HEVs have been described as being responsible for respiratory syndromes, including EV-68, EV-C104, EV-C105, EV-C109, and EV-C116. Most of these HEV strains belong to HEV-C species [5]-[8], [12], [13], [23]–[26]. The aim of this study was to characterise the genome of two novel HEV-C strains, EV-C117 and EV-C118, identified in patients with CAP. These new viruses were both identified during the course of the CAP-PRI study, but were not epidemiologically related: EV-C117 was detected in a respiratory sample of a Lithuanian child hospitalised because of CAP [14], [15], and EV-C118 was identified in respiratory samples of two Israeli Bedouin children hospitalised because of CAP and acute otitis media [16], [17].

Both EV-C117 and EV-C118 have the typical enterovirus genome organisation [2]. They both have a *cis*-acting replication element (CRE) in the form of a small RNA hairpin in the coding region of protein 2C, which has been shown to be critical for RNA replication in picornaviruses [27]. In addition, a series of genomic features essential to virus replication was identified in the genome [2], [28], [29]. The 5′UTR sequences of EV-C117 and EV-C118 were related to those reported for EV-C104, EV-C105 and EV-C109, and were slightly shorter than those of other HEV-A to HEV-D [5], [12], [13]; they also belong to a different clade from those of the two classic clades reported by Santti and others [30]–[32]. Our data support the hypothesis of an ancestral recombinant origin of the 5′UTR [5], [6], [12], [13], but the significance of the emergence of this unconventional 5′UTR is still unknown.

Intra- and intertypical recombinations are common within HEV-C [32]–[35]. However, no evidence of recombination was observed in the coding region of the EV-C117 and EV-C118 strains, and this is supported by the congruent phylogeny across their entire genomes. The results of the phylogenetic, Simplot and bootscan analyses indicate that recombination was not the main mechanism of evolution for EV-C117 and EV-C118, which strengthems the hypothesis of the monophyletic origin of the coding regions, as has been observed in other HEV-C [36].

VP1 is the most exposed capsid protein and is usually more variable than other virus genes. The VP1 sequences of EV-C117 and EV-C118 fulfilled the molecular typing criteria proposed by Oberste *et al*. to define a new HEV type (75% VP1 nucleotide identity and 85 or 88% VP1 amino acid identity) [3], [4]. There was a striking difference in variability within the VP1 sequences: the β-sheet motifs were conserved, and the greatest variability was observed in the loop motifs and both the N- and C-terminus regions. These findings are in line with previous descriptions of the distribution of VP1 variability [37], [38].

The phylogenetic trees constructed using the sequences of the P1, P2 and P3 regions were comparable with those constructed using the sequences of the entire genome. EV-C117 was closely related to EV-C104, whereas EV-C118 grouped with EV-C105 and EV-C109 in all of the trees (excluding P1). The HEV-C fell into three different groups, with group III consisting entirely of HEV strains with shorter 5′UTR sequences that have been identified during the last five years, including EV-C117 and EV-C118. As the recent detection of a number of HEVs belonging to this new group may be the result of increased surveillance and improved molecular methods, additional isolates that evolved along lineages that are independent of the original reference strain are needed in order to clarify the relationships within the group. Finally, phylogenetic analyses showed that group III is divided into two subgroups (IIIa and IIIb) with high bootstrap support, although the 5′UTR sequences from the two subgroups clustered together. These findings suggest the stringent conservation of a 5′UTR containing a secondary structure essential for virus replication, or the silent circulation of these viruses followed by viral evolution due to the accumulation of point mutations in the coding region.

In conclusion, our findings characterise the complete genome of two new HEV-C. Analysis of the variation of the EV-C117 and EV-C118 sequences within the enterovirus species revealed greater sequence homology in the 5′UTR, less homology in the P1 region, and greater homology in the P2 and P3 region. The phylogenetic analysis showed the emergence of a new group within HEV-C, divided into two subgroups. Nucleotide and amino acid identity in VP1 sequences have been established as useful criteria for assigning new HEV types, but analysis of the complete P1 region improves the resolution of their identification and characterisation.
